# The Status of *EGFR* Modulates the Effect of miRNA-200c on *ZEB1* Expression and Cell Migration in Glioblastoma Cells

**DOI:** 10.3390/ijms22010368

**Published:** 2020-12-31

**Authors:** Lisandra Muñoz-Hidalgo, Teresa San-Miguel, Javier Megías, Eva Serna, Silvia Calabuig-Fariñas, Daniel Monleón, Rosario Gil-Benso, Miguel Cerdá-Nicolás, Concha López-Ginés

**Affiliations:** 1 INCLIVA, Clinic Hospital of Valencia, 46010 Valencia, Spain; lisandramh@gmail.com (L.M.-H.); Jose.m.cerda@uv.es (M.C.-N.); 2 Department of Pathology, Faculty of Medicine and Dentistry, University of Valencia, 46010 Valencia, Spain; Teresa.Miguel@uv.es (T.S.-M.); Silvia.Calabuig@uv.es (S.C.-F.); daniel.monleon@uv.es (D.M.); rosario.gil-benso@uv.es (R.G.-B.); Concha.lopez@uv.es (C.L.-G.); 3 Department of Physiology, Faculty of Medicine and Dentistry, University of Valencia, 46010 Valencia, Spain; eva.serna@uv.es; 4 Centro de Investigación Biomédica en Red en Cáncer (CIBERONC), 28029 Madrid, Spain; 5 Molecular Oncology Laboratory, Fundación Hospital General Universitario de Valencia, 46014 Valencia, Spain; 6 TRIAL Mixed Unit, Centro de Investigación Príncipe Felipe-Fundación para la Investigación del Hospital Ge-neral Universitario de València, 46012 Valencia, Spain

**Keywords:** glioblastoma, EGFR amplification, miR-200c, ZEB1, cell migration

## Abstract

Migration of glioblastoma cells into surrounding tissue is one of the main features that makes this tumor incurable. We evaluated whole-genome miRNA expression profiling associated with different EGFR amplification patterns in 30 cases of primary glioblastoma. From the 64 miRNAs that showed differential expression between tumors with a high level of EGFR amplification and tumors without EGFR amplification, 40% were related with cell migration, being miR-200c the most differentially expressed between these two groups. We investigated the effect of miR-200c on ZEB1 expression and cell migration in an in vitro transfection model with a miR-200c mimic, a miR-200c inhibitor and siRNA targeting EGFR in three short-term cultures with different levels of EGFR amplification obtained from resected glioblastomas. The cell culture with the highest EGFR amplification level presented the lowest miR-200c expression and the status of EGFR modulated the effect of miR-200c on ZEB1 expression. Silencing EGFR led to miR-200c upregulation and ZEB1 downregulation in transfected cultures, except in the presence of high levels of EGFR. Likewise, miR-200c upregulation decreased ZEB1 expression and inhibited cell migration, especially when EGFR was not amplified. Our results suggest that modulating miR-200c may serve as a novel therapeutic approach for glioblastoma depending on EGFR status.

## 1. Introduction

Glioblastoma (GBM) is among the most frequent and most malignant primary brain tumor types in adults. Despite the use of aggressive treatment regimens, the prognosis is still poor and most of the patients die within one year of diagnosis [1]. While GBM rarely metastasizes, its cells migrate from the tumor mass and infiltrate the surrounding brain tissue. Because of this highly invasive nature, complete resection of the entire tumor during surgical intervention is impossible and therapeutic actions are ineffective [1,2,3]. This points to the migratory pattern of GBM cells as the major obstacle to combatting them with the current therapies and the need to find new strategies to overcome this obstacle [4,5]. The epidermal growth factor receptor gene (*EGFR*) is often amplified, mutated and overexpressed in primary GBMs. There is a strong variation in frequency and level of *EGFR* amplification in GBM. Different studies report values between 35% and 70% for *EGFR* amplification or overexpression [6,7,8]. This disparity on the reported frequencies is probably due to the diversity in methodologies [8,9,10]. According to *EGFR* copy-number, three GBM groups have been reported. First, GBMs with a high level of *EGFR* amplification in the form of double minutes (dmin). Additionally, this group frequently presents copies of the mutant variant of the gene, *EGFRvIII*. Second, GBMs with a low level of *EGFR* amplification as insertions into different loci on chromosome 7. Third, GBMs without *EGFR* amplification [8,11]. Although these *EGFR* copy-number groups have different biological implications, their clinical relevance is still poorly understood.

MicroRNAs (miRNAs) are a type of noncoding RNAs of approximately 20–30 nucleotides in length that play important roles as regulators of mRNA translation [12]. Aberrant expression patterns in several miRNAs, especially miR-15b, miR-21, miR-221/222 and miR-296, are associated with the pathogenesis and progression of GBM [13,14,15,16,17,18].

In the present work, we studied a set of miRNAs whose expression levels were significantly altered depending on the amplification status of *EGFR* in a cohort of 30 human GBMs. Our analyses were performed in order to find correlations between 64 miRNAs associated with different *EGFR* amplification patterns and their roles in biological processes related to cancer. Since cell migration is a key biological process related to GBM aggressiveness, we focused our study on this process, finding a subset of miRNAs with promigratory and antimigratory effects. From them, miR-200c exhibited the most significant differential expression in tumors with and without *EGFR* amplification.

The MiR-200 family plays different roles in the progression of many epithelial neoplasms and its dysregulation can decrease cell−cell adhesion, enhance migratory capabilities, and promote epithelial-mesenchymal transition (EMT), a process that triggers cellular mobility and subsequent dissemination of cancer cells [19,20,21,22]. However, the astrocytic origin of GBM precludes its association with the concept of a straight EMT, as these tumors rarely express E-cadherin [23,24]. Although a proper EMT is not at play in gliomas, some evidence suggests a role for EMT-promoting transcription factors (EMT-TFs) in the genesis and progression of primary GBM, similar to epithelial tumors [25,26,27].

From the many transcription factors which induce EMT, the repressor zinc-finger E-box binding homeobox (ZEB) family of transcription factors exhibit a double-negative feedback loop with the miR-200 family cluster that regulates EMT in different cell systems [21,22]. A member of the ZEB family, ZEB1 has been implicated in the processes of primary GBM tumor initiation, cell invasion and resistance to the drug temozolomide [28,29]. This transcription factor was shown to be part, together with Sox2 and Olig2, of a transcriptional network that drives gliomagenesis [30]. ZEB1 is able to modulate the expression of genes which are considered mediators of tumor cell migration and invasion, like E-cadherin, a cell membrane calcium-dependent glycoprotein responsible for cell−cell adhesion [22,31], or the guanine nucleotide exchange factor Prex1, which has been shown to be important for the invasion of GBM cells in vivo [32].

Although some studies report the influence of miR-200c in GBM, it is still unclear how this miRNA family is involved in this mechanism. The interrelationships between EGFR and miR-200 family are poorly understood, and EGF/EGFR signaling has been suggested to regulate the aggressiveness of glioma cells by modulating the miR-200 family member’s expression [15,33,34]. In addition, most of these studies are performed on established cell lines that do not show *EGFR* amplification, since this amplification is lost as the cells adapt to the in vitro conditions.

In this study, we also analyze miR-200c expression, its potential regulatory role on *ZEB1* expression and its effect on cell migration in an in vitro model with human GBM cells that display different *EGFR* amplification status. We performed transfection procedures, overexpressing or inhibiting miR-200c as well as silencing *EGFR* in an established cell line and three primary cultures of GBM, two of them with *EGFR* amplification, with one of those containing the *EGFR* mutant variant. This allowed us to explore, for the first time, the associations between EGFR, miRNA200c and ZEB1 and to discover new mechanistic insights about their potential interactions.

## 2. Results

### 2.1. Whole-Genome miRNA Analysis and Cell Migration Biological Analysis

**Figure 1:**
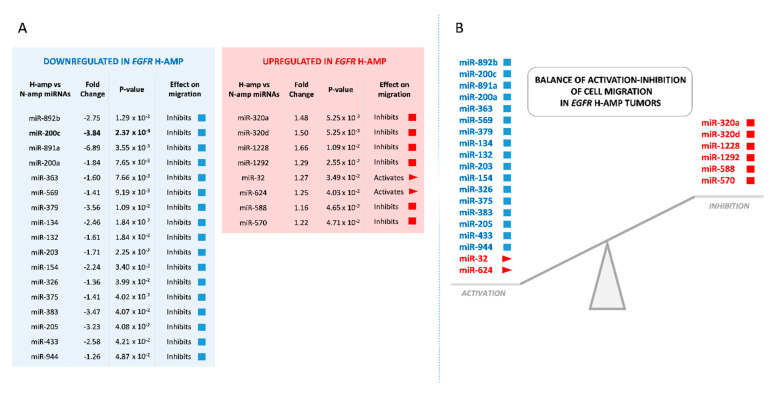
MiRNAs implied in cell migration that are differentially expressed in *EGFR* H-amp and N-amp tumors. (**A**) MiRNAs downregulated and upregulated in H-amp with respect to N-amp, ordered by statistical significance (*p ≤ 0.05*). (**B**) Scheme showing the balance between miRNAs whose expression would activate migration and miRNAs whose expression would inhibit migration in H-amp samples.

In a previous work of our group, 30 primary GBMs were analyzed by FISH in tissue microarrays and categorized according to the level of *EGFR* amplification: high level *EGFR* amplification (H-amp, 13 cases), low level *EGFR* amplification (L-amp, 7 cases) and no *EGFR* amplification (N-amp, 10 cases) [15]. Here, after miRNA expression microarray analysis by one-way ANOVA among these different *EGFR* amplification groups, 64 well-annotated miRNAs with significant expression changes between samples of *EGFR* H-amp and N-amp groups were detected. Then, we studied the possible correlations between the known functions of these 64 miRNAs and their roles in biological processes related to GBM progression. Interestingly, almost 40% of the miRNAs (25 of the 64) were found to participate in modulating cell migration. From these 25 miRNAs, seventeen were downregulated in H-amp samples and eight were upregulated. Only two would act promoting migration and the other 23 inhibiting it (Figure 1A). The expression of promigratory and antimigratory miRNAs showed a clear tendency towards the activation of migration in H-amp tumors. The two promigratory miRNAs were significantly more expressed in H-amp GBMs compared to N-amp, whereas most of the antimigratory miRNAs (17 from the total 25) were less expressed in H-amp tumors compared to N-amp tumors (Figure 1B). This imbalance supports the idea that H-amp tumors would display a higher migration activity when compared with tumors without *EGFR* amplification, at least considering miRNA expression patterns.

### 2.2. Downregulation of miR-200c in H-amp Tumors May Promote Cell Migration

Since we observed that the expression patterns of miRNAs in our cohort were consistent with the activation of migration in *EGFR* H-amp tumors, we decided to further study the role of the most prominent miRNA on H-amp cells migration compared to the other *EGFR* amplification groups: L-amp and N-amp.

Two antimigratory miRNAs stood out by their differential expression between H-amp and N-amp groups; miR-200c and miR-891a. Both molecules were downregulated in H-amp tumors and showed the highest fold change values from the 25 miRNAs, but differences were more significant in the case of miR-200c, with a *p*-value of 2.37 × 10^−3^ (Figure 1A). For this reason, we decided to stablish an in vitro model of human GBM cells with different *EGFR* amplification status in order to further study miR-200c behavior, its role on the transcription factor *ZEB1* expression and its effect on cell migration.

### 2.3. In Vitro Model of EGFR Amplification: Cell Culture Characterization

Short-term cell cultures were isolated from tumors obtained from patients undergoing GBM resection. We selected three short-term GBM cell cultures from our laboratory with differences for *EGFR* amplification: HC-444 culture without *EGFR* amplification (N-amp), HC-534 with a low *EGFR* amplification level (L-amp) and HC-466 with high *EGFR* amplification level (H-amp) (Supplementary Material Table S1). We have also worked with the commercial cell line U-118, without *EGFR* amplification (N-amp).

**Figure 2:**
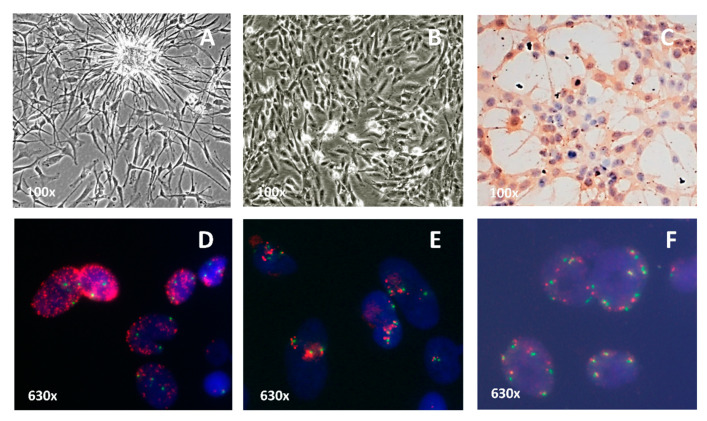
Characterization of the short-term cell cultures. (**A**) Cells with an elongated morphology and cytoplasmic expansions in HC-466 culture (100×). (**B**) Cells with a variable morphology and many mitoses in HC-534 culture (100×). (**C**) Positive expression of the GFAP protein in HC-444 culture (100×). (**D**) Tumor cells with a high level of *EGFR* amplification in HC-466 culture (630×). (**E**) Tumor cells with low level of *EGFR* amplification in HC-534 culture (630×). (**F**) Cells without *EGFR* amplification in HC-444 culture (630×). FISH analysis was carried out with the dual probe for EGFR (red) and centromere of chromosome 7 (green). The FISH micrographs are representative from three independent experiments.

Cultures grew in a monolayer and exhibited variable growth patterns and heterogeneous cellular morphology (Figure 2A,B). Immunohistochemical analysis showed they all expressed GFAP in the cytoplasm (Figure 2C). EGFR protein was overexpressed in HC-466 (level 3) and in HC-534 (level 2) cultures.

Cytogenetic patterns were studied by karyotyping the three primary cultures and the established cell line U-118. In all cases, the maximum number of metaphases obtained ranged from 3 to 25. The chromosomal numbers of the different GBM cell cultures varied from hypodiploid to hyperdiploid range, with frequent presence of polyploidy. Chromosome 7 trisomy was present in all the cultures and chromosomal rearrangements were very common, but their heterogeneity made it impossible to detail them in some of the karyotypes (Supplementary Material Table S2). The positivity for GFAP and the cytogenetic characteristics demonstrate the glial and tumor profile of the three primary cultures.

Cultures were categorized based on their *EGFR* status according to FISH analysis: in the HC-466 culture, the proportion of cells with H-amp *EGFR* was higher than 50%, with a large number of gene copies in each cell (more than 20 copies) (Figure 2D). In the HC-534 culture, the fraction of cells with L-amp *EGFR* ranged from 5% to 20% and showed a small number of *EGFR* copies in each cell (between 3 and 6 copies) (Figure 2E). N-amp cell cultures showed two copies per nuclei (Figure 2F). In addition, MLPA analysis showed the presence of the mutant variant *EGFRvIII* only in the H-amp HC-466 culture cells (Supplementary Material Table S2).

### 2.4. EGFR, miR-200c and ZEB1 Expression in Cell Cultures

**Figure 3:**
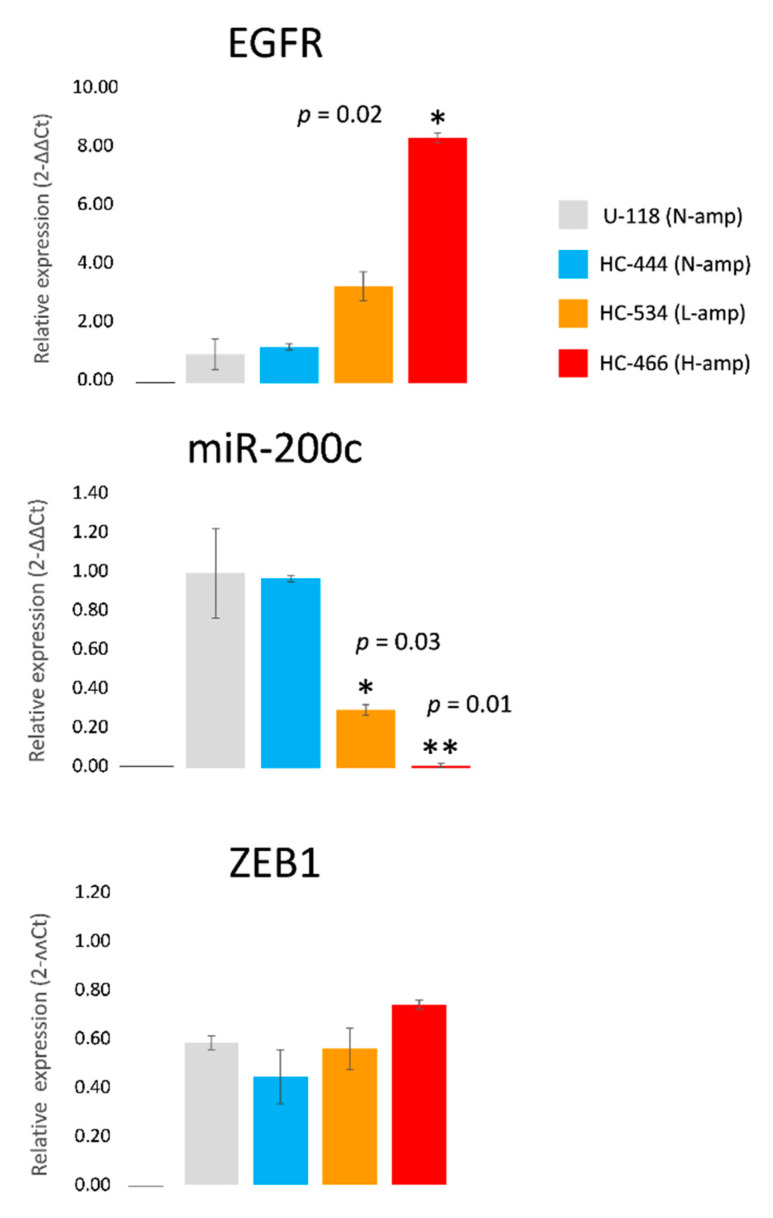
*EGFR* mRNA, miR-200c and *ZEB1* mRNA expression in the cultures with different levels of *EGFR* amplification. Results were normalized to actin (*EGFR* and *ZEB1*) or U66 (miR-200c) housekeeping genes. Changes in RNA expression are reported as the mean and standard error with respect to the nonamplified *EGFR* group using the 2-∆∆Ct method. Four independent experiments were performed. Statistical analyses were performed using one-way analysis of variance, followed by Dunnett’s t-test for multiple comparisons. Significant expression changes (*p* ≤ 0.05 and *p* ≤ 0.01) with respect to U-118 are marked with * and **, respectively.

The expression levels of *EGFR*, miR-200c and *ZEB1* were analyzed in the four cell cultures by RT-PCR. A relationship between the level of *EGFR* amplification and its expression was observed; cultures with *EGFR* amplification showed increased EGFR protein expression which reached statistical significance in the HC-466 culture (fold change: 8.47 ± 0.17, *p* = 0.02) when compared to N-amp cultures (Figure 3). In *EGFR* amplified cultures (HC-466 and HC-534), miR-200c was downregulated with respect to the cultures without *EGFR* amplification (U-118 and HC-444), these differences being statistically significant (Figure 3). This result is concordant with the miR-200c downregulation observed previously in H-amp tumors compared to N-amp tumors (Figure 1A). In contrast, ZEB1 protein expression showed a nonstatistically significant tendency to increase in the *EGFR* amplified cultures compared with the N-amp HC-444 culture (Figure 3).

### 2.5. Transfection Efficiency and Cell Viability in GBM Transfected Cultures

Transfection efficiency ranged from 85 to 95% in all the cases. The four cultures transfected with the miR-200c mimic showed fold increases in miR-200c expression that ranged from 4 × 10^4^ to 10 × 10^4^, compared with the miRNA mimic negative control, whereas transfection with miR-200c inhibitor showed strong decreases of more than two thirds of the control values in all the cases, compared with the miRNA mimic negative control.

**Figure 4:**
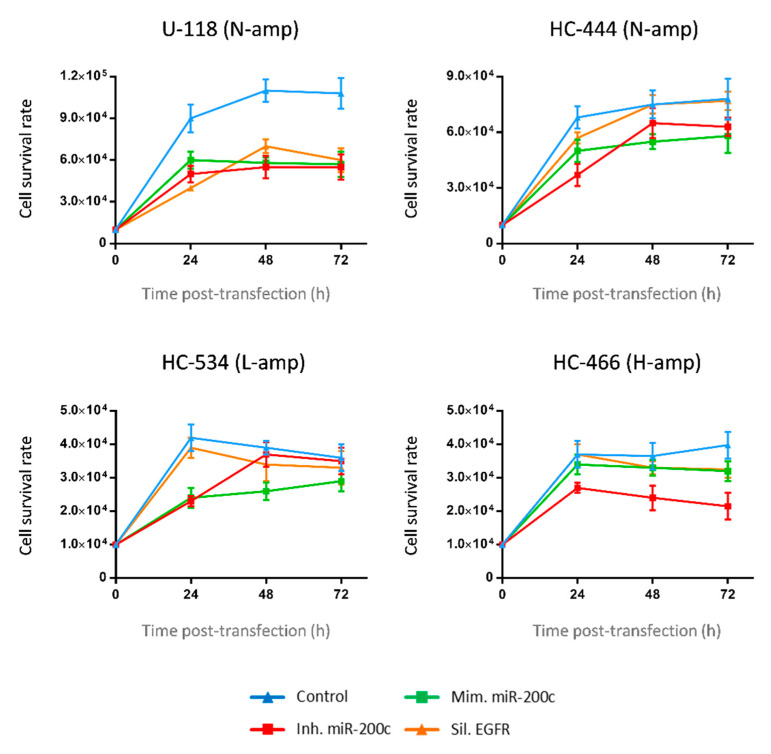
Cell viability. Cell viability study by luminescence in the four transfected cell cultures (U-118, HC-444, HC-534 and HC-466) with miR-200c mimic, miR-200c inhibitor and *EGFR* silencer conditions, with respect to control (h: hours). The points for each curve represent the mean values ± SDs of three independent experiments. Statistical analyses were performed using one-way analysis of variance, followed by Dunnett’s t-test for multiple comparisons.

Cell viability of transfected cultures was determined in three experimental conditions: miR-200c inhibition, miR-200c overexpression and *EGFR* silencing. Control (nontransfected) cells showed the highest viability in each culture, without significant changes among the different conditions (Figure 4). Noteworthy, cell viability remained very close to the control in *EGFR*-silenced cells, especially in those cultures with *EGFR* amplification (Figure 4).

### 2.6. MiR-200c Modulates ZEB1 and EGFR Expression in Cultures without EGFR Amplification

To determine the effect of miR-200c on *ZEB1* expression, we overexpressed or knocked down miR-200c by transfection with miR-200c mimic or miR-200c inhibitory oligonucleotides in primitive GBM cell cultures and in U-118 cell line.

**Figure 5:**
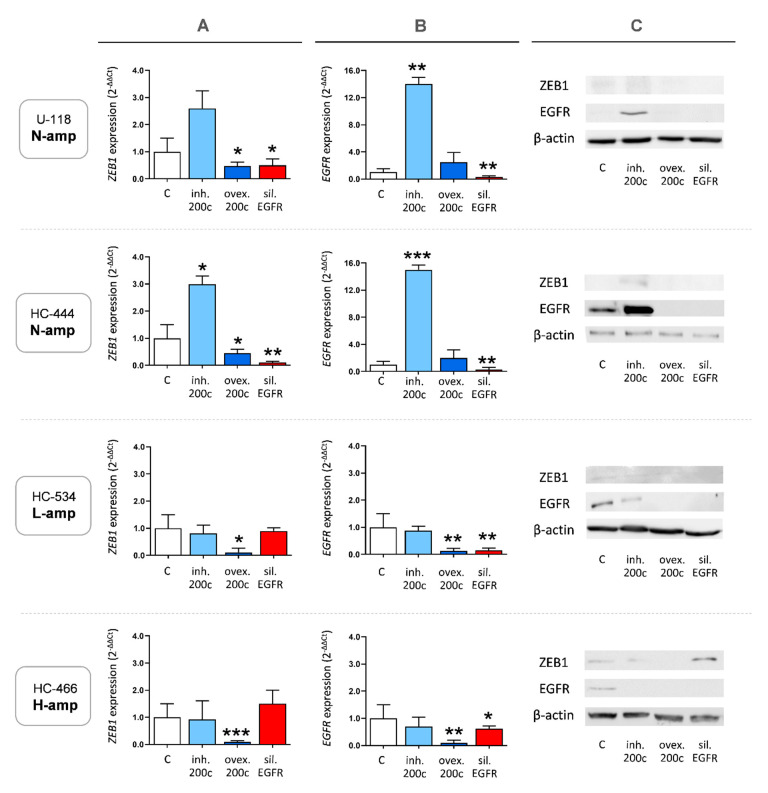
*ZEB1* and *EGFR* expression analyses. (**A**) *ZEB1* mRNA expression by qRT-PCR after performing cultures with four experimental conditions: without transfection (control), miR-200c inhibition, miR-200c overexpression and *EGFR* silencing. (**B**) *EGFR* mRNA expression by qRT-PCR after performing cultures with the four aforementioned experimental conditions. (**C**) ZEB1 and EGFR protein expression by Western blot after performing cultures with the four aforementioned experimental conditions. Changes in mRNA expression are reported as the mean and standard error with respect to the nontransfected culture using the 2-∆∆Ct method. All the experiments were performed three times. Statistical analyses were performed using one-way analysis of variance, followed by Dunnett’s t-test for multiple comparisons. Significant expression changes (*p* ≤ 0.05, *p* ≤ 0.01 and *p* ≤ 0.001) are marked with *, ** and ***, respectively. Results were normalized to the actin housekeeping gene.

First, we examined the expression changes in N-amp cultures. As shown in Figure 5, both N-amp cultures (U-118 and HC-444) showed similar *ZEB1* and *EGFR* expression patterns when miR-200c was modulated. MiR-200c overexpression downregulated *ZEB1* mRNA and protein expression in N-amp cultures, while inhibition of miR-200c produced the opposite effect (Figure 5A,C). Silencing *EGFR* reduced *ZEB1* expression, this effect being significant in HC-444 cells. With respect to *EGFR* expression, while inhibition of miR-200c produced a significant increase, overexpression of miR-200c had no significant consequences on *EGFR* mRNA or protein levels with respect to control cells (Figure 5B,C). This may be due to the low basal *EGFR* expression in N-amp control cultures, which would make it difficult to distinguish small expression differences.

### 2.7. Overexpression of miR-200c Diminishes ZEB1 and EGFR Expression in Cultures with EGFR Amplification

Cultures with *EGFR* amplification (L-amp HC-534 and H-amp HC-466) showed a coincident behavior when miR-200c was overexpressed or inhibited. MiR-200c overexpression downregulated significantly *ZEB1* mRNA and protein expression. Instead, and differentially to the results observed in N-amp cells, inhibition of miR-200c did not produce a clear effect on *ZEB1* expression (Figure 5A,C), maybe as a consequence of other unknown interactions related with the complex phenotype of GMB cells with *EGFR* amplification. Silencing *EGFR* did not change *ZEB1* expression in HC-534 cells, but produced a slight increase in HC-466 culture that was significant at the protein level (Figure 5A,C). This may have been due to the expression of the *EGFRvIII* mutant variant in these H-amp cells.

### 2.8. MiR-200c Upregulation in GBM Cell Cultures Inhibits Cell Migration

**Figure 6:**
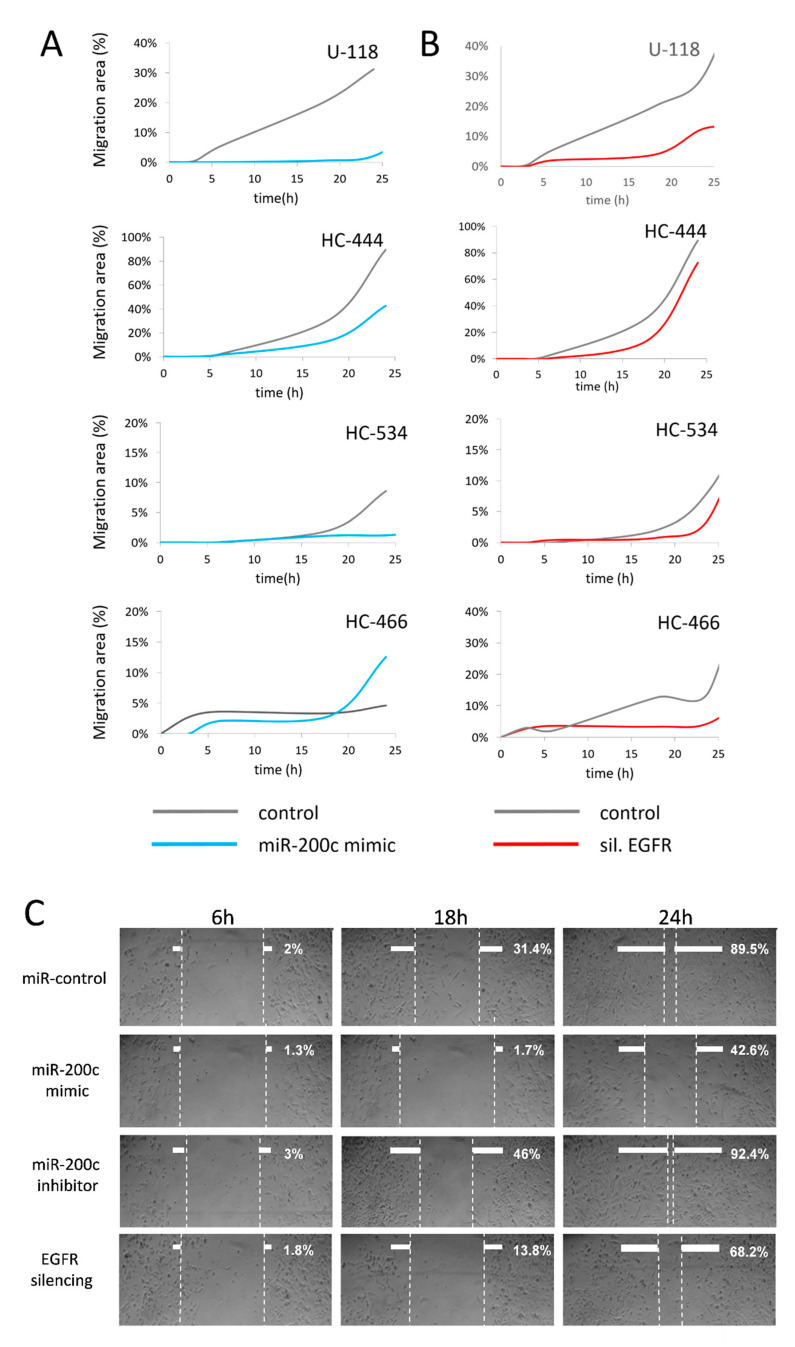
Cell migration assays. (**A**) Effect of miR-200c mimic transfection on cell migration in the four cultures at different times. (**B**) Effect of EGFR siRNAi transfection on cell migration in the four cultures at different times. (**C**) Wound healing assay in HC-444 cells. The wound healing assay was conducted 6, 18 and 24 h after transfection in: control cells, cells overexpressing miR-200c, cells with miR-200c inhibition and cells with silenced EGFR. Pictures show the results in HC-444 culture. All the experiments were performed three times. Graphs and micrographs show one representative experiment from three.

The effects of miR-200c on cell growth and migration in control, miR-200c overexpression, miR-200c inhibition and *EGFR* silencing conditions were examined. Cell migration was determined via scratch wound-healing assays and the areas of migration were measured at 6, 18 and 24 h post-transfection. Only U-118 and HC-466 control cells presented detectable migratory activity between 0 and 3 h in all conditions (Figure 6A). When miR-200c was overexpressed, cell migration levels were lower compared to control cultures at 6, 8 and 24 h post-transfection except for HC-466, which showed increased cell migration at 18 h post-transfection (Figure 6A). In contrast, when miR-200c was inhibited, cell migration increased with respect to controls at different time points: HC-444 and HC-534 from 6 h, U-118 from 18 h and HC-466 from 24 h (data not shown).

In this experimental setting, *EGFR* silencing resulted in decreased cell migration which was observed for U-118 and HC-444 cells from 6 h post-transfection, for HC-534 cells from 18 h and at different time points for HC-466 cells (Figure 6B). The decrease in cell migration was more evident when *EGFR* amplification was absent (Figure 6C shows culture HC-444 results) or in cultures with low *EGFR* amplification, and this decrease was not observed or at least was not so evident in HC-466 cultures with amplified and mutated *EGFR* (Figure 6A). Notably, analysis of cell migration effects in the different cultures revealed that those cultures with low *EGFR* amplification or without it responded homogeneously to both miR-200c overexpression and *EGFR* silencing, resulting in a stable cell migration inhibition.

## 3. Discussion

GBM is characterized by extensive, diffuse tumor cell infiltration, and this growth pattern is a major determinant in therapeutic failure. There is evidence for dysregulated miRNAs differentially expressed in GBMs compared to low grade and/or anaplastic astrocytomas. This suggests that altered miRNA expression plays an important role in gliomagenesis and glioma progression, and that some of these miRNAs might be useful as prognostic markers [35,36]. Since these molecules have demonstrated a potential role in many biological processes related to GBM progression, we studied the role of a series of miRNAs in 30 human primary tumors, categorized according to their *EGFR* amplification levels, as previously described [10,15].

Cell migration of GBM, one of the key processes that influences the fatal outcome of this tumor [4], has been related with miRNA expression alterations [4,37,38,39]. In our study, the differential expression of 64 miRNAs associated with cell migration between *EGFR* H-amp and N-amp tumors indicated an increased activation of this tumorigenic process in GMBs with *EGFR* amplification. Thus, miRNA expression patterns in H-amp GBMs would be a relevant contributor, together with many other elements, of the increased aggressiveness of this subgroup of tumors compared to GBMs without *EGFR* amplification. Several miRNAs stood out by their significant expression differences between *EGFR* groups, supporting an important role on activation of cell migration in H-amp GBMs. Some of them were, in order of relevance (according to *p*-value and fold change parameters), the antimigratory miRNAs: miR-200c, miR-891a, miR-892b or miR-200a.

The role of miRNA-200c has been described in different epithelial tumors, and the loss of its expression is associated with disease progression in different neoplasms, including breast, bladder, lung and ovarian cancers [36,40,41]. Several studies suggest that some miR-200 family members, including miR-200b and miR-141, are significantly downregulated in glioma tissues, and that this dysregulation correlates with the pathological grade of gliomas [42,43]. Low expression levels of miRNA-891a in breast cancer have been recently associated with low distant metastasis-free survival, implying that the expression of miR-891a-5p is a potential prognosis marker for metastatic human breast cancers [44]. MiR-892b inhibits proliferation, migration and invasion in bladder cancer cell lines [45]. MiR-200a has been related with the inhibition of migration in non-small cell lung cancer [46] and pancreatic ductal adenocarcinoma [47], but may promote cell proliferation, migration, and invasion in colorectal carcinoma [48].

From all the studied miRNAs, the prominent expression differences of miR-200c between H-amp and N-amp groups prompted us to develop an experimental in vitro transfection model that showed the effects of overexpressing or abolishing this miRNA on cell migration.

Studies in primary tumors, as well as in cell lines, have shown that miR-200c is involved in migration and in EMT process. EMT is induced by different transcription factors, including ZEB1/ZEB2. There is a strong inverse relationship between the expression of miR-200 family members and ZEB1/ZEB2 expression [22,49]. Like their role in epithelial tumors, transcription factors associated to EMT also promote cellular invasion in primary GBM in an epithelial-to-mesenchymal(-like) transition manner, as a real EMT cannot be associated with this tumor with astrocytic origin [50]. However, there is little knowledge about how ZEB1 is regulated by miR-200c in GBM [29,51,52].

The contribution of *EGFR* overexpression on EMT and epithelial-to-mesenchymal(-like) transition activation remains unresolved. The inhibition of *EGFR* expression strongly suppressed *ZEB1* expression in primary carcinoma tumors and cell lines [53]. In these tumors, EGFR promoted EMT by upregulating *ZEB1* and its blockade was sufficient to obstruct the EMT induced by TGF-β [53]. EGF/EGFR signaling regulates the aggressiveness of anaplastic thyroid cell lines by modulating miR-200 expression [33]. Although a straight EMT does not occur in GBM and these interactions have been scarcely studied, both “The Cancer Genome Atlas (TCGA)” data and our results showed that miR-200c expression is lower in samples with *EGFR* amplification [34].

In the present study, we have demonstrated statistically significant associations between *EGFR* amplification and miR-200c expression in GBM cell cultures and cell lines. Cells with the highest levels of *EGFR* amplification showed the lowest levels of miR-200c (Figure 3). By using a miR-200c mimic and a miR-200c inhibitor, we explored the impact of miR-200c levels on *ZEB1* expression and cell migration in association with *EGFR* status. All transfected cultures showed *ZEB1* expression blockade when miR-200c was overexpressed, independently of *EGFR* amplification status.

**Figure 7:**
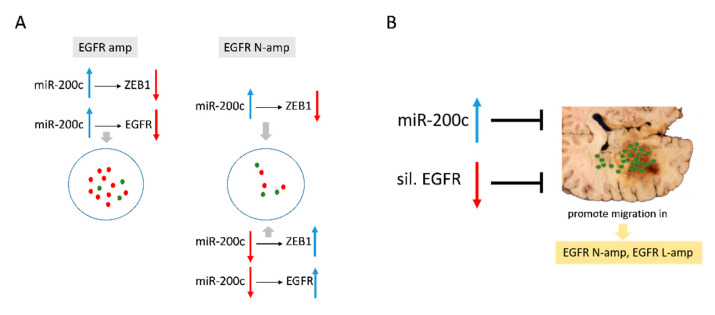
(**A**) MiR-200c modulates *ZEB1* and *EGFR* expression. (**B**) Effects of miR-200c expression and *EGFR* silencing on cell migration.

However, miR-200c inhibition resulted in increased *ZEB1* expression only in cultures without *EGFR* amplification, suggesting a potential alternative mechanism for *ZEB1* modulation in *EGFR* amplified cells (Figure 7A). The phenotype of *EGFR* amplified cells, with unknown complex biochemical interactions, could explain the effects observed with miR-200c inhibition on *ZEB1* and *EGFR* expression. In addition, the high presence of copies of the *EGFRvIII* mutant variant in the H-amp HC-466 culture may influence its behavior and make *EGFR* silencing fail, at least partially.

Taken together, the present results provide the first evidence of a mechanistic link between miR-200c and EGFR, which is dependent on the amplification of the *EGFR* gene.

Previous works aiming to determine the role of miR-200c on GBM cellular migration are very scarce and are limited to studies with cell lines [14,42,43]. However, the feedback regulation between miR-200c and ZEB1 suggests that *ZEB1* expression may be associated with tumor cell infiltration [29]. In this work, in addition to a cell line, we have used three GBM short-term cell cultures. Short-term cultures are composed of cells very close in phenotype and genotype to the original tumor cells. This represents a major advantage for exploring the cell biology of the tumor with respect to established cell lines and other in vitro models. Here we report that overexpression of miR-200c abolished cell migration in all the tested GBM cell cultures except that with the highest *EGFR* amplification and with the presence of the *EGFRvIII* mutant variant (HC-466). Indeed, inhibition of miR-200c resulted in increased cell migration, albeit at different times in the different cultures. Likewise, while published work evaluating the role of *EGFR* silencing in cellular migration provided inconclusive data [27], here we show that *EGFR* silencing resulted in decreased cell migration, which was more evident in those cultures where *EGFR* amplification was absent or low. The mechanistic implications of this observation are rather complex. Silencing by siRNA was effective in all cell cultures with high, low or no *EGFR* amplification, however, our data suggests a potential mechanism, associated to *EGFR* amplification and alternative to miR-200c expression, for *ZEB1* regulation. This may similarly impact cell motility and would explain our cell migration results.

To the best of our knowledge, this is the first study showing the role of several miRNAs whose differential expression between GBMs with *EGFR* amplification and without it to indicate an activation of cell migration in tumors from the first group.

It is also the first study to show that miR-200c is involved in cell migration in a manner dependent on *EGFR* amplification status in GBM cells. Our results both in human GBMs and in in vitro GBM short-term cultures identify miR-200c as an important player and potential new molecular target by modulating the migratory capacity of these tumors. Furthermore, we have also provided evidence that the status of *EGFR* modulates the effect of miR-200c on *ZEB1* expression, and the upregulation of miR-200c inhibits cell migration in the absence of high *EGFR* amplification (Figure 7B).

Our study demonstrates molecular links between miR-200c, ZEB1 and EGFR in GBM cells and their impact on migratory activity. MiRNAs and their targets may become valuable tools in the characterization, diagnosis, prognosis and treatment of GBM. Specifically, miR-200c modulation, especially in tumors with low or no *EGFR* amplification, could be a potential therapeutic tool against GBM infiltration and recurrences.

## 4. Materials and Methods

### 4.1. Tumor Samples

Tumor samples were collected from 30 patients with a diagnosis of primary GBM from the Department of Neurosurgery of the Hospital Clínico Universitario de Valencia. Patients gave written informed consent for participating in the study, which was approved by the Institutional Ethics Committee of the University of Valencia and Clinic Hospital of Valencia, with approval registration code 2010/140 (30-06-2011, Ley 14/2007 de Investigación Biomédica).

Samples were treated and analyzed as previously described [15].

### 4.2. MiRNA Expression Analysis in GBM Tumors

Total RNA from frozen tissue of different groups was extracted using a mirVANA miRNA Isolation Kit (Ambion, Austin, TX, USA). The quality and integrity of total RNA was determined by capillary electrophoresis using the Bioanalyzer 2100 (Agilent Technologies, Santa Clara, CA, USA). Only RNA extracts with RNA integrity number values ≥ 6 underwent a further analysis. RNA was used in a GeneChip miRNA Array containing 46,228 probe sets representing 6703 miRNA sequences from the Sanger miRNA database (V.11) and an additional 922 sequences of Human snoRNA and scaRNA from the Ensembl database and snoRNABase (Affimetrix, Santa Clara, CA, USA) was used for microarray analysis according to manufacturer’s instructions.

Data files (.CEL) were analyzed with Partek Genomic Suite 6.6 software (Partex Inc., St. Louis, MO, USA). Input files were normalized with a robust multichip average (RMA) algorithm for gene array and analyzed with the web-based miRNA QC Tool software (www.affymetrix.com).

### 4.3. Cell Cultures and Treatment

We worked with three short-term GBM cultures named HC-444 (N-amp), HC-534 (L-amp) and HC-466 (H-amp), obtained directly from resected human GBMs and subcultured less than five times in our laboratory. Informed consent was obtained from the patients and approval for the study herein reported was provided by Institutional Ethics Committee of the University of Valencia and Clinic Hospital of Valencia, with approval registration code 2010/140 (30-06-2011, Ley 14/2007 de Investigación Biomédica).

We also used the U-118 GBM human cell line from the American Type Culture Collection (ATCC, Manassas, VA, USA). This study was conducted in accordance with the provisions of the Declaration of Helsinki and was approved by the Institutional Ethics Committee of the University of Valencia and Clinic Hospital of Valencia, with approval registration code 2010/140 (30-06-2011, Ley 14/2007 de Investigación Biomédica).

Primary cell cultures were obtained from primary GBM tumor tissue samples by disaggregation using 0.02% collagenase type II (Sigma-Aldrich, St. Louis, MO, USA) and seeding cells in 25-cm^2^ tissue culture flasks (Nunc, Roskilde, Denmark). Cells were subcultured using 0.25% trypsin–ethylenediaminetetraacetic acid (EDTA) (Gibco BRL, Grand Island, NY, USA) once they reached confluence, and used at passages 2–4 from the primary cell cultures. All the cell cultures were grown in RPMI 1640 medium (Gibco BRL, Grand Island, NY, USA) supplemented with 10% fetal bovine serum (Gibco BRL, Grand Island, NY, USA), 1% L-glutamine and antibiotics (50 U/mL penicillin and 50 µg/mL streptomycin) in a humidified atmosphere with 5% CO_2_ at 37 °C.

### 4.4. Immunophenotypical Analysis

Nunc Lab-Tek Chamber Slide System (Thermo Scientific, Waltham, MA, USA) was used for immunocytochemistry studies. Cells were cultured at a concentration of 0.02 × 10^6^ cells/well. After culture, slides were washed with PBS and cells were fixed with methanol/acetone for 5 min. Monoclonal antibodies against glial fibrillary acidic protein (GFAP) and EGFR (wild-type EGFR and its deletion-mutant variant EGFRvIII) were used (Dako, Glostrup, Denmark). EGFR expression was evaluated according to the number of stained cells and the staining intensity: 0 (no staining), 1 (light or focal staining), 2 (moderate staining, present in 50% to 75% of the sample) and 3 (strong staining, present in more than 75% of the sample). Scores between 0 and 1 were defined as absence of overexpression, whereas scores of 2 and 3 were defined as overexpression. Two experienced pathologists analyzed each slide.

### 4.5. Conventional Cytogenetics and Fluorescence in Situ Hybridization (FISH)

Cells obtained from the first short-term culture passages were exposed to colcemid (0.02 µg/mL) for 2 h at 37 °C and harvested routinely. Metaphasic chromosomes were G-banded using a conventional trypsin-Giemsa technique and karyotypic analyses were performed according to the 2020 International System for Cytogenetic Nomenclature [54].

To evaluate the *EGFR* status, dual color FISH analysis was performed on cultured cells using the LSR EGFR Spectrum Orange/CEP-7 Spectrum Green Probe from Vysis (Abbott Laboratories, Downers Grove, IL, USA; Cat. No. 32-191053). Hybridizations were performed according to the manufacturer’s instructions and the nuclei were counterstained with DAPI. Fluorescent signals were detected using a Leica DM400B photomicroscope (Leica, Madrid, Spain) equipped with an appropriate filter set. Signals were counted in 100–150 cell nuclei. In each case the mean signal numbers for *EGFR* and the control *CEP-7* probe were calculated and used to determine the *EGFR*/*CEP-7* ratio. *EGFR* was amplified when the *EGFR*/*CEP-7* signal ratio was > 2 [55].

### 4.6. Multiplex Ligation-Dependent Probe Amplification (MLPA)

DNA was obtained from cultured cells with QIAmp DNA Mini Kit (Qiagen, Inc., Valencia, CA, USA) and multiplex ligation-dependent probe amplification (MLPA) was performed to determine the number of copies of the mutant variant III of *EGFR* (*EGFRvIII*) by establishing a *EGFRvIII* ratio between the average ratio for exons 2–7 probes and the average ratio of exons 1, 8, 13, 17 and 22 probes. SALSA MLPA kits were used according to the manufacturer’s instructions (MRC-Holland, Amsterdam, Netherlands). Fragments were separated with a sequencer ABI 310 (Applied Biosystems Inc., Foster City, CA, USA). Analysis was performed with the Coffalyser software (MRC-Holland). Ratios under 0.8 units were related with the *EGFRvIII* deletion variant.

### 4.7. RNA Extraction and Quantitative Real-Time PCR

MirVANA miRNA Isolation Kit was used to extract and purify total RNA from cell cultures (Ambion, Austin, TX, USA). The integrity and quality were tested by a Bioanalyzer 2100 (Agilent Technologies, Santa Clara, CA, USA). Only extracts with integrity number values ≥ 6 were considered for subsequent analysis.

Expression of mRNA and miRNA was studied by RT-PCR with a TaqMan MicroRNA Reverse Transcription Kit (Applied Biosystems). MiR-200c was amplified with TaqMan probes (hsa-miR-200c, REF. 002300, Applied Biosystems). TaqMan Universal Master Mix II was used for Real Time-PCR analysis (Applied Biosystems). Taqman probes Hs01023894_m1, Hs01076078_m1 and Hs00232783_m1 were used for *EGFR* and *ZEB1* amplification, respectively (Applied Biosystems). Real Time-PCR analysis was performed with TaqMan Gene Expression kit (Applied Biosystems). Relative expression differences among groups were expressed using cycle threshold (Ct) values as follows: Ct values were first normalized with RNU66 (REF: 001002) for mir200c (REF: 002300) and ACTB (REF: Hs99999903_m1) for EGFR and ZEB1 of the same sample. Dunnett’s t test was applied for comparisons between groups. Results were considered statistically significant if *p*-value *p* ≤ 0.05.

### 4.8. Western Blot Analysis

Total protein was extracted from cell cultures using an extraction buffer containing 10 mM tris-HCl (pH 7.5), 0.25 M sucrose, 5 mM EDTA, 50 mM sodium chloride, 30 mM sodium pyrophosphate, 50 mM sodium fluoride and 100 mM sodium orthovanadate. A protein inhibitor cocktail (Sigma-Aldrich, ref: P8340) was added just before use in a concentration of 5 mg/mL. Protein concentrations were determined with bovine serum albumin as reference standard using the Foling−Lowry protein assay (Sigma-Aldrich). Protein lysates (40 μg) were resolved in 12% denaturing sodium dodecyl sulfate-polyacrylamide gels, transferred to nitrocellulose membranes (Bio-Rad Laboratories, Hercules, CA, USA) using Trans Blot Turbo TM Transfer, and incubated with primary antibodies against ZEB1 (ZEB1 monoclonal antibody, M01), and EGFR, both from Abnova (Taipei, Taiwan). Signals were detected by incubation with a secondary antibody (Jackson Immuno Research Inc., West Grove, PA, USA) labelled with SuperSignal West Pico chemiluminescent reagent kit (Fisher Scientific, Waltham, Massachusetts, USA) and revealed with a chemiluminescent Image Quant Las 4000 (GE Healthcare, Chicago, IL, USA).

### 4.9. Cell Transfection

Short-term cell cultures and U-118 GBM cell line were transiently transfected with a miR-200c mimic, a miR-200c inhibitor and a siRNA targeting EGFR. The scrambled control oligonucleotides were purchased from Thermo Fisher Scientific. The positive control mimic miR-1 was used for transfection experiments where mRNA overexpression was required. Overexpression of miR-1 caused silencing of PTK9 mRNA, which indicated the efficiency of the transfection assay. The let-7c positive control downregulated HMGA2 mRNA. Inhibition of miR-let-7 was an internal transfection control and resulted in double the HMGA2 mRNA expression, as in the control expression, indicating the high efficiency of the transfection assay. Culture cells were transfected using Lipofectamine 2000 reagent (RNA MAX, Thermo Fisher Scientific) following the manufacturer’s instructions. To check miR-200c expression after transfection, total RNA was isolated from cells 48 h after transfection and miR-200c expression levels were examined by RT-PCR.

### 4.10. Luminescence Cell Viability Assay

After transfection, cells were seeded into 96-well plates at a density of 10,000 cells per well. Cell viability was measured daily at 0, 24, 48 and 72 h post-transfection using a CellTiter-Glo Luminescent Assay (Promega Biotech Ibérica, Madrid, Spain) and a VICTOR multilabel plate reader 2030 (Perkin Elmer, Madrid, Spain).

### 4.11. Wound Healing Assay

Tumor cell migration capacity was assessed using the CytoSelect Wound Healing Assay Kit (Bionova, Madrid, Spain), which measures the speed at which a monolayer of cultured and confluent cells moves into a gap or wound. Images were taken at 0, 6, 18 and 24 h, and measured using Image Pro Plus 6.0 software (Leica DMIL LED).

### 4.12. Statistical Analyses 

The MiRNAs expression results in primary GBMs were analyzed with Partek Genomic Suite 6.6 (Partek Inc., St. Louis, MO, USA). A one-way ANOVA was performed and nonparametric Kruskal−Wallis test was used to select miRNAs differentially expressed among the tumors with high level of *EGFR* amplification (H-amp), low level of *EGFR* amplification (L-amp) and those with no *EGFR* amplification (N-amp) with at least a significance level of *p* ≤ 0.05.

In vitro results are presented as mean ± SDs. Statistical analyses were performed using one-way analysis of variance, followed by Dunnett’s t-test for multiple comparisons. Results were considered statistically significant from *p* ≤ 0.05.

## Data Availability

Data is contained within the article or supplementary material.

## Supplementary Materials

Supplementary materials can be found at https://www.mdpi.com/1422-0067/22/1/368/s1.
