# Supplementary material for: The Status of EGFR Modulates the Effect of miRNA-200c on ZEB1 Expression and Cell Migration in Glioblastoma Cells
Source: Int J Mol Sci. 2020 Dec 31;22(1):368. doi: 10.3390/ijms22010368 (PMC7795155; doi:10.3390/ijms22010368)
Supplement: Supplementary file 1 [file ijms-22-00368-s001.zip › Table S1.docx]

**Supplementary Material Table S1**. Clinical findings in the patients with GBM.

| **Patient** | **Culture** | **Age/Sex** | **Location** | **Size**  **(cm)** | **KI** | **Treatment** | **Survival (months)** |
| --- | --- | --- | --- | --- | --- | --- | --- |
| 1 | HC-444 | 54/M | T | 4.0 | 100 | RT+CH | 5 |
| 2 | HC-534 | 31/F | F | 2.0 | 70 | RT | 5 |
| 3 | HC-466 | 55/F | CC | 4.0 | 70 | CH | 4 |

Sex: male (M), female (F). Location: frontal (F), temporal (T), corpus callosum (CC). KI: Karnosky index. Treatment: radiotherapy (RT), chemotherapy (CH). Survival: all cases were *exitus* at the end of the present study.
