# Supplementary material for: The Status of EGFR Modulates the Effect of miRNA-200c on ZEB1 Expression and Cell Migration in Glioblastoma Cells
Source: Int J Mol Sci. 2020 Dec 31;22(1):368. doi: 10.3390/ijms22010368 (PMC7795155; doi:10.3390/ijms22010368)
Supplement: Supplementary file 1 [file ijms-22-00368-s001.zip › Table S2.docx]

**Supplementary Material Table S2.** Genetic features of cell cultures.

| **Cultures** | **FISH/*EGFR*** | ***EGFRvIII*** | **Karyotype** |
| --- | --- | --- | --- |
| U-118 | N-amp | - | +7, t(11;5), t(8q;4), t(19;18). Polyploidy |
| HC-444 | N-amp | - | 47-55,XY),+7 der(11),+18 Polyploidy |
| HC-534 | L-amp | - | 42-44,XX,+7,-10,-19,-22 |
| HC-466 | H-amp | + | +7,dmin |
